# Radiographic entheseal lesions of the pelvic region are more prevalent in radiographic axSpA than in age- and sex-matched controls and are associated with more severe spinal disease

**DOI:** 10.1007/s10067-025-07345-8

**Published:** 2025-01-30

**Authors:** F. R. Wink, T. Diemel, S. Arends, A. Spoorenberg

**Affiliations:** 1 Rheumatology and Clinical Immunology, University of Groningen, University Medical Center Groningen, Groningen, The Netherlands; 2 Rheumatology, Medical Center Leeuwarden, Henri Dunantweg 2, 8934 AD Leeuwarden, The Netherlands; 3 Geriatric Medicine, Radboud University Medical Center, Nijmegen, The Netherlands

**Keywords:** Axial spondyloarthritis, Disease severity, Entheseal lesions, Enthesitis, Pelvis, Radiographs

## Abstract

**Objectives:**

In axial spondyloarthritis (axSpA), entheseal involvement is common, which contributes significantly to disease burden and may also lead to structural damage. Although radiographs of the pelvis are widely available in axSpA, information on entheseal damage and associated characteristics are lacking. Therefore, we assessed the prevalence of radiographic entheseal lesions at the pelvic region in radiographic (r-) axSpA compared with controls and explored associations with patient and disease characteristics.

**Methods:**

Pelvic radiographs of 167 consecutive r-axSpA patients were randomized with 100 pelvic radiographs from age- and sex-matched controls. Radiographs were blinded for patient information and sacroiliac joints and bilaterally evaluated for erosions/cortical irregularities, enthesophytes, and calcifications by two trained readers at the greater and lesser trochanter, os ischium, and iliac crest.

**Results:**

Entheseal lesions were observed in 127 (76%) of r-axSpA patients and 58 (58%) controls. R-axSpA patients showed significantly more (bilateral) entheseal lesions than controls at all entheseal sites. Most lesions were found at the os ischium, erosions/cortical irregularities were most prevalent, and calcification was the most specific lesion in r-axSpA. Patients with lesions were significantly older, had longer symptom duration, and more severe spinal radiographic damage than patients without lesions. Enthesophytes were found significantly more often in patients with body mass index (BMI) ≥ 25.

**Conclusion:**

Structural entheseal lesions observed at pelvic radiographs are not specific but occur often in r-axSpA patients. Treating physicians should keep in mind that these entheseal lesions are associated with more severe axial disease and high BMI which may be relevant for treatment decisions.

**Table:** 

**Key Points** • Pelvic radiographic entheseal lesions are significantly more prevalent in r-axSpA patients than in controls. • Radiographic entheseal lesions are associated with longer symptom duration and more spinal radiographic damage. • Pelvic enthesophytes are found significantly more often in r-axSpA patients with BMI ≥ 25.

## Introduction

The sacroiliac joints and the spine are predominantly affected in axial spondyloarthritis (axSpA). Peripheral manifestations such as arthritis, dactylitis, and enthesitis are reported in up to 66% of the patients [1]. Enthesitis is the most frequently reported peripheral SpA feature and is associated with higher disease activity and more severe outcome [2, 3]. Also, burden of disease is higher and quality of life lower in axSpA patients with clinical enthesitis [4]. Nevertheless, clinical evaluation of enthesitis is difficult, which may lead to underdiagnosis of enthesitis [2]. Therefore, it is important to recognize entheseal involvement and to take this into account for treatment decisions [2, 3, 5].

Enthesitis is not specific for SpA and can also occur in healthy subjects. In the normal population, enthesitis is most often caused by mechanical overloading, such as repetitive work or sports activities, in which mostly a single entheseal insertion is involved. Generally, it recovers spontaneously taking rest. In contrast, enthesitis in SpA is frequently chronic and affecting more entheseal insertions at the same time [2, 6]. 

In SpA, the exact pathophysiological mechanism of enthesitis is not clear yet. Probably, a lower threshold for triggering entheseal inflammation plays a role [7]. Histopathology is the “gold standard” for the diagnosis of enthesitis; however, this is not feasible in clinical practice and research. Therefore, entheseal involvement in (ax)SpA is often assessed by clinical examination. Especially for research, standardized clinical enthesitis assessments have been developed, e.g., the Maastricht Ankylosing Spondylitis Enthesitis Score (MASES) and Spondyloarthritis Research Consortium of Canada (SPARCC) index [8, 9]. Besides local tenderness at the entheseal insertion during palpation, limited other inflammatory characteristics are present, which makes it difficult to discriminate between inflammatory and non-inflammatory involvement [10].

Imaging techniques may be of additional value. Conventional radiographs may show signs of entheseal structural damage, such as erosions/irregularity, enthesophytes, and calcifications [10]. Ultrasound (US) and magnetic resonance imaging (MRI) can provide information on signs of entheseal inflammation, such as increased thickness and Doppler activity on US and bone marrow edema or soft tissue edema on MRI, but structural damage is limited visible [10–12]. Although US and MRI are becoming more accessible, they are more time-consuming and expensive.

Correlations of entheseal involvement assessed with physical examination and inflammatory entheseal lesions on US and MRI in axSpA are low (between 45–95% and 57–21%, respectively) [11, 12]. Therefore, reported prevalence rates of enthesitis in axSpA depend on the type of assessment used and vary between 21% (clinical assessment) and 95% (US lesions). Also, in healthy subjects, entheseal lesions are regularly found with US (up to 73%), most often enthesophytes, whereas erosions were infrequently found [13]. The presence of these lesions in healthy subjects was associated with older age, male sex, higher body mass index (BMI), and physical activity level and smoking [14].

Several studies in radiographic axSpA (r-axSpA) have shown that the presence of enthesophytes on US is associated with radiographic spinal damage, reflected by higher modified Stoke Ankylosing Spondylitis Spine Score (mSASSS) and more syndesmophytes [15–17]. In r-axSpA patients, the presence of enthesophytes of the Achilles tendon on US was associated with male sex, whereas the presence of clinical enthesitis (SPARCC index) was found more often in female patients [2, 15]. BMI was also associated with the presence of enthesophytes in axSpA patients [15, 16].

Concerning the location of entheseal involvement, the insertion of the Achilles tendon and plantar fascia are considered as typical locations for enthesitis in axSpA. However, multiple studies showed that entheseal involvement can also be present at other entheseal insertions of the upper and lower extremities [6, 11, 12, 18]. Interestingly, in two whole-body (WB-) MRI studies, the entheseal locations of the pelvis were most frequently affected in axSpA [6, 11]. As far as we know, there are no studies on entheseal involvement of the pelvis and hip region on conventional radiographs in r-axSpA in comparison to healthy subjects, which is remarkable because these radiographs are available in almost all axSpA patients.

Therefore, the aim of this study was to assess the prevalence and the specificity of three types of radiographic lesions (erosions/irregularity, enthesophytes, and calcifications) associated with entheseal involvement at the hip and pelvic region in r-axSpA patients in comparison to age- and sex-matched controls. Furthermore, to explore if these radiographic entheseal lesions are associated with patient and disease characteristics.

## Patients and methods

### Patients and controls

Between November 2004 and December 2010, consecutive r-axSpA patients from the Groningen Leeuwarden axSpA (GLAS) cohort, before starting TNF-α inhibitors (TNFi) and with an available anterior–posterior (AP) pelvic radiograph at baseline, were included in this study. All patients were diagnosed with r-axSpA, fulfilled the modified New York criteria, and were ≥ 18 years old. We selected r-axSpA patients for this explorative study since they have more severe (axial) disease than non-radiographic (nr-) axSpA patients and, therefore, have a higher probability to have structural entheseal lesions.

Furthermore, 100 AP pelvic radiographs from age- and sex-matched controls who visited the emergency department before March 2018 were randomly selected from the radiology department archives of the University Medical Center Groningen (UMCG), based on frequency matching of 10-year age cohorts for males and females. The exact reason for the pelvic radiographs was unknown; inflammatory back pain is however no indication to visit the emergency department in Dutch medical centers.

The GLAS cohort including the addendum of this project were approved by the local ethics committees of the UMCG and MCL (RTPO 364), and all patients gave written informed consent according to the Declaration of Helsinki.

### Clinical assessments

All patients were clinically evaluated before start of treatment with TNFi within the standardized GLAS cohort protocol [19]. Disease activity was assessed with the Axial Spondyloarthritis Disease Activity Score (ASDAS), Bath Ankylosing Spondylitis Disease Activity Index (BASDAI), and C-reactive protein (CRP) [20, 21]. Physical function was assessed with the Bath Ankylosing Spondylitis Functional Index (BASFI) [22]. Clinical enthesitis was assessed at 26 entheses. This includes all entheses of the MASES supplemented with the following entheses: medial and lateral femurcondyl, ischial tuberosities, greater trochanter and plantar fascia (all at both sides), and C1/C2, C7/Th1, and Th12/L1.

Spinal and hip radiographic damage was scored by two independent and trained readers blinded to patient characteristics using the mSASSS and the Bath Ankylosing Spondylitis Radiology Hip Index (BASRI-hip), respectively [23, 24].

### Radiological assessment

The anterior–posterior view of pelvic radiographs was used for assessment. Prior to the assessment, relevant literature and pelvic radiographs of r-axSpA patients were studied and discussed with all investigators to determine the scoring method, in which locations and types of entheseal lesions were designated for this study [25, 26]. Radiological features of enthesopathy are characterized by bone loss (erosions) and new bone formation (enthesophytes, calcifications); therefore, we chose to focus on these type of lesions. As discrimination between definite erosions and “only” irregularity of the bone appeared difficult, both lesions were combined into one type of lesion.

**Fig. 1:**
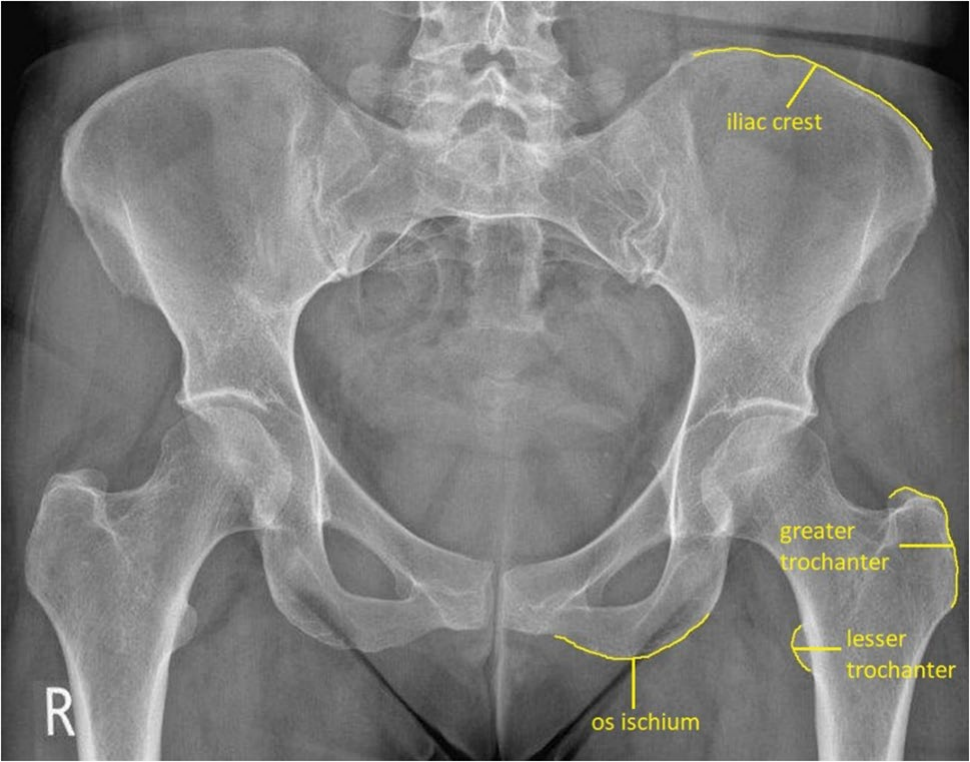
Anatomical locations evaluated in the present study

The entheseal locations on the pelvis radiograph selected for evaluation were the greater trochanter (attachment of gluteus muscles and piriformis), lesser trochanter (attachment of psoas major and iliacus), os ischium (attachment of quadratus femoris, externus obturatorius, adductor magnus, semitendinosus, semimembranosus, biceps femoris and gemellus superior), and iliac crest (attachment of inguinal ligament, tensor fasciae latae and sartorius), for all both left and right sides (Fig. 1) [27, 28].

**Fig. 2:**
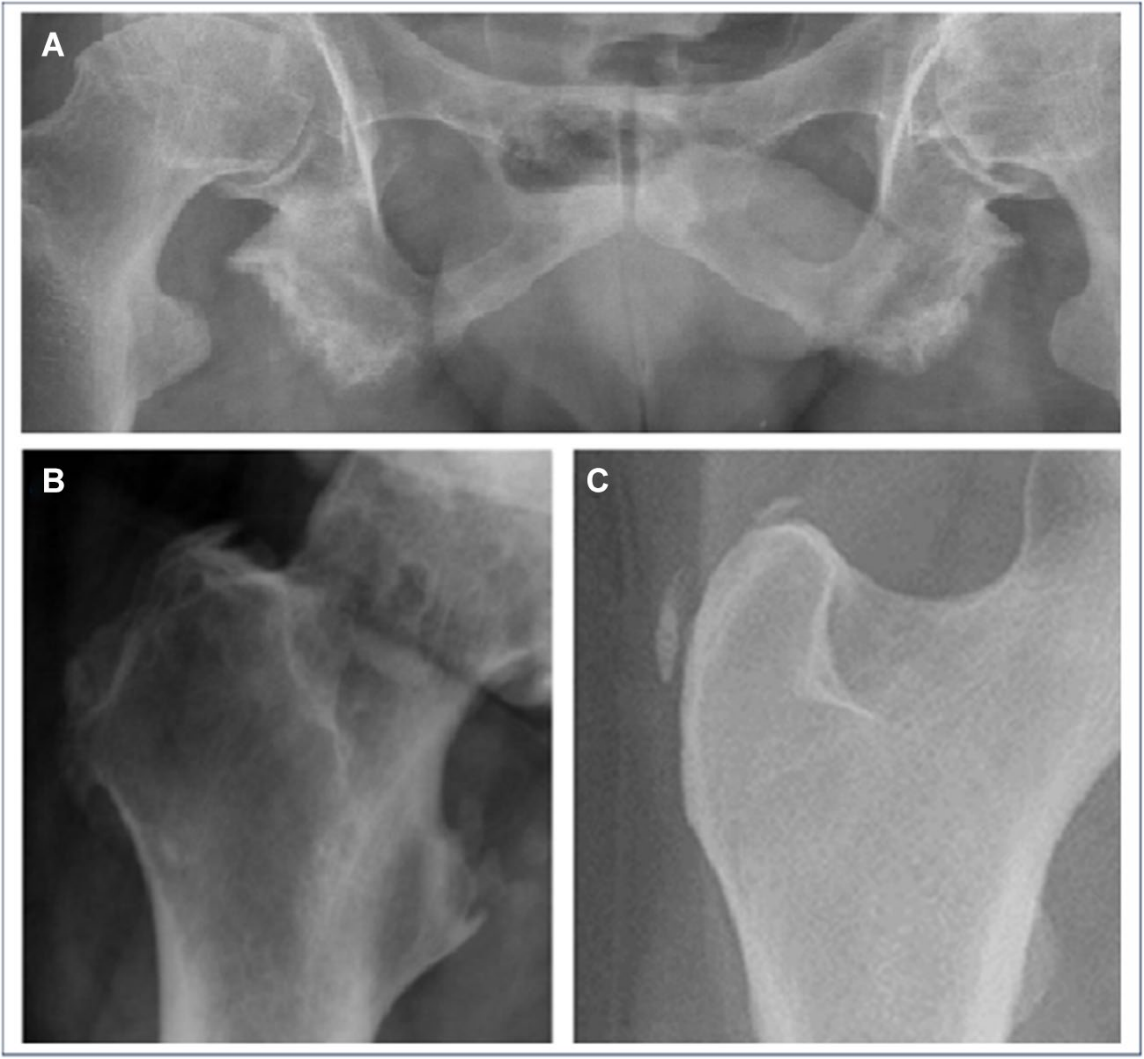
Example lesions scored at different entheseal locations. **A** Erosions/cortical irregularities at the os ischium. **B** Enthesophyte at the greater and lesser trochanter. **C** Calcifications at the greater trochanter

The different types of radiographic lesions scored were erosions/cortical irregularities, enthesophytes, and calcifications. Erosions and/or cortical irregularities were defined as focal loss of cortical bone at the entheseal insertion and irregular edge of the insertion at the cortical bone, respectively. Enthesophytes were defined as bony proliferation connected with the entheseal insertion and projected in the direction of the alignment of the ligament. Calcifications were defined as bony proliferations not attached to the bone, but orientated in the entheseal region (Fig. 2). All anomalies were scored as absent (0) or present (1). In case an entheseal location was not completely visible, it was scored as missing. A normal pelvis radiograph was used as reference when a possible entheseal lesion was doubted.

The two readers (FW and TD) had several training sessions to gain experience with the scoring method. After each training session, 35 different pelvic radiographs were scored individually and lesions were compared and discussed in detail. After three training sessions, the scores of the 35 radiographs showed moderate to good inter-observer agreement with Cohen kappa between 0.47 and 0.88. Furthermore, intra-observer agreement was fair to good with Cohen kappa between 0.32 and 0.74. Absolute agreement of all lesions was > 85%.

For the scoring, all radiographs of r-axSpA patients and controls were randomized and individual information was removed from the radiographs. Importantly, the sacroiliac joints of all radiographs were blinded. Radiographs were scored independently by the two readers. Only lesions with absolute agreement between both readers were used for further statistical analysis.

### Statistical analysis

Prevalence rates were expressed as the percentage of patients or controls with anomalies present. Furthermore, the percentages for type of lesion and location were reported separately. Normally distributed data were reported as mean and standard deviation (SD) and non-normally distributed data as median and interquartile range (IQR). Specificity for the presence of entheseal lesions in r-axSpA patients was calculated in total and for each type of radiographic entheseal lesion and location.

The association between the number of radiographic entheseal lesions at the eight different locations and the number of tender entheses was analyzed using the Spearman correlation coefficient. Chi-square or Fisher’s exact test, independent samples *t*-test, and Mann–Whitney *U* test were used when appropriate to compare patient and disease characteristics between patients with and without radiographic entheseal lesions. Univariable and multivariable logistic regression analyses were used to analyze which characteristics were independently associated with the presence of radiographic entheseal lesions. *P*-values ≤ 0.05 were considered statistically significant.

Statistical analysis was performed using the PASW Statistics 28 (SPSS, Chicago, IL, USA).

## Results

In total, 167 r-axSpA patients were included in this study. Mean age was 43 years (SD ± 11), 70% was male, median symptom duration was 16 years (IQR 7–24), and 80% was HLA-B27 positive. Patients had high disease activity, with mean ASDAS of 3.7 ± 0.8, mean BASDAI of 6.1 ± 1.6, and median CRP of 12 (4–21). All patient characteristics are presented in Table 1.

**Table 1:** Characteristics of the total group of r-axSpA patients and comparing r-axSpA patients with and without entheseal lesions

	All patients (*n* = 167)	r-axSpA with entheseal lesions (*n* = 127)	r-axSpA without entheseal lesions (*n* = 40)
Sex (male) (*n*, %)	117 (70)	95 (75)	22 (55)
Age (years)	43.0 ± 10.9	45.2 ± 10.1*	35.1 ± 9.5
Symptom duration (years)	16 (7–24)	18 (10–24)*	7.5 (4–20)
HLA-B27 + (*n*, %)	133 (80)	98 (77)	35 (87)
BMI	26.2 (23.8–29.0)	26.9 (24.2–29.1)	25.0 (22.8–28.9)
BMI ≥ 25 (*n*, %)	73 (44)	56 (34)	17 (10)
BMI ≥ 30 (*n*, %)	19 (11)	14 (8)	5 (3)
History of psoriasis (*n*, %)	9 (5)	9 (7)	0
History of IBD (*n*, %)	15 (9)	13 (10)	2 (5)
History of uveitis (*n*, %)	47 (28)	39 (31)	8 (20)
History of peripheral arthritis (n, %)	60 (36)	44 (35)	16 (41)
History of hip involvement (n, %)	34 (20)	27 (6)	7 (19)
Tender entheses (range 0–28)	3 (0–6)	3 (0–6)	3 (0–5)
ASDAS	3.7 ± 0.8	3.7 ± 0.8	3.7 ± 0.9
BASDAI (range 0–10)	6.1 ± 1.6	6.1 ± 1.6	6.1 ± 1.5
CRP (mg/L)	12 (4–21)	12 (4–18)	14 (6–28)
BASFI (range 0–10)	5.8 (4.1–7.1)	5.8 (4.2–7.2)	5.2 (3.1–6.5)
Radiologic damage			
mSASSS total score (range 0–72)	6.5 (1.0–18.7)	8.7 (2.5–24.7)*	1.0 (0.0–6.8)
mSASSS cervical spine	3.0 (0.5–9.6)	4.5 (1.2–14.1)*	0.5 (0.0–3.8)
mSASSS lumbar spine	2.0 (0–9.8)	4.0 (0.0–14.4)*	0.0 (0.0–2.0)
BASRI-hip (range 0–4)	0.0 (0.0–0.0)	0.0 (0.0–0.5)	0.0 (0.0–0.0)
BASRI-hip ≥ 2	12 (7%)	11 (9%)	1 (3%)

Values are presented as mean ± SD or median (IQR) unless otherwise indicated

*r-axSpA* radiological axial spondyloarthritis, *n* number, *HLA-B27* + human leukocyte antigen B27 positive, *BMI* body mass index, *IBD* inflammatory bowel disease, *BASDAI* Bath Ankylosing Spondylitis Disease Activity Index, *ASDAS* Axial Spondyloarthritis Disease Activity Score, *CRP* C-reactive protein, *BASFI* Bath Ankylosing Spondylitis Functional Index, *mSASSS* modified Stoke Ankylosing Spondylitis Spinal Score, *BASRI-hip* Bath Ankylosing Spondylitis Radiology-hip

*p < 0.005R

The overall prevalence of radiographical entheseal lesions of the pelvic region was 127 (76%) for r-axSpA patients and 58 (58%) for controls, which was significantly different (*p* < 0.001) (Table 2). A much larger total number of lesions was found in r-axSpA patients than in controls, 344 versus 124, respectively. In r-axSpA patients, these lesions were significantly more often bilateral (58% vs 38%; *p* = 0.002). The median number of lesions was 2 (IQR 0–4) in r-axSpA patients and 0.5 (0–2) in controls, which was also significantly different (*p* < 0.001). Specificity for total number of lesions at all locations was 40% in r-axSpA. For the individual locations and types of lesions, prevalence was varying between 37 and 70% in r-axSpA (Table 2).

**Table 2:** Number, prevalence and specificity of radiographic entheseal lesions specified for type of lesion total and per location at the pelvis and hip region in r-axSpA patients (*n* = 167) versus age- and sex-matched controls (*n* = 100)

	Number of lesions		Prevalence		Specificity
	CS	r-axSpA	CS	r-axSpA	
All lesions	122	344	58% (58/100)	76% (127/167)**	40%
Erosions/irregularities	83	232	51% (51/100)	72% (120/167)**	49%
Calcification	2	11	2% (2/100)	5% (9/167)	98%
Enthesophyte	37	101	21% (21/100)	31% (52/167)	79%
Greater trochanter					
All lesions	8	50	4% (4/100)	21% (34/161)**	96%
Erosions/irregularities	2	24	1% (1/100)	11% (18/161)**	99%
Calcification	0	9	0% (0/100)	5% (8/160)	100%
Enthesophyte	6	17	4% (4/100)	9% (14/160)	96%
Lesser trochanter					
All lesions	5	32	5% (5/96)	15% (24/159)*	95%
Erosions/irregularities	1	13	5% (5/96)	15% (24/159)	95%
Calcification	1	1	1% (1/95)	0.6% (1/158)	99%
Enthesophyte	3	18	3% (3/96)	9% (14/158)	97%
Os ischium					
All lesions	97	218	53% (53/100)	66% (108/163)*	47%
Erosions/irregularities	80	195	49% (49/100)	65% (106/163)*	51%
Calcification	1	1	1% (1/99)	0.6% (1/163)	99%
Enthesophyte	16	22	12% (12/99)	9% (14/163)	88%
Iliac crest					
All lesions	12	44	9% (8/94)	19% (28/151)*	91%
Erosions/irregularities	0	0	0% (0/93)	0% (0/150)	-
Calcification	0	0	0% (0/93)	0% (0/150)	-
Enthesophyte	12	44	9% (8/94)	19% (28/151)*	91%

Values are presented as valid percentage of patients

*r-axSpA* radiographic axial spondyloarthritis, *n* number, *CS* controls

*p < 0.05; **p < 0.005

### Type of lesion and location

Erosions/cortical irregularities were the most prevalent lesions found in both r-axSpA patients and controls, which were significantly more frequently observed in r-axSpA (72% vs 51%; *p* < 0.001) (Table 2). At the greater trochanter and os ischium, these erosions/irregularities were found significantly more often in patients than in controls. At the iliac crest, no erosions/irregularities were found in both patients and controls. Bilateral erosions/irregularities were seen in 63% of r-axSpA patients compared with 33% of controls, which was significantly different (*p* < 0.001).

The prevalence of calcifications was low in both patients and controls (5% vs 2%). At the greater trochanter, calcifications were most often present and observed more often in r-axSpA patients, with a trend toward significancy (5% vs 0%, *p* = 0.053) (Table 2). Only one r-axSpA patient showed a bilateral calcification.

Enthesophytes were observed frequently in both r-axSpA patients and controls (31% vs 21%), which was only significantly different at the iliac crest (19% vs 9%; *p* = 0.037) (Table 2). Although bilateral enthesophytes were more often present in patients than in controls, this was not statistically significant (16% vs 9%, *p* = 0.063). Both calcifications and enthesophytes were the most specific radiographic entheseal lesions in r-axSpA patients (98% and 79%, respectively).

At all four entheseal locations, r-axSpA patients showed significantly more lesions than controls (Table 2). In both r-axSpA patients and controls, most entheseal lesions were found at the os ischium (66% vs 53%; *p* = 0.037) and the lowest number of lesions were found at the lesser trochanter (15% vs 5%) (Table 2). The greater and lesser trochanter were the most specific location for all types entheseal of lesions in r-axSpA, varying from 47 to 100%.

At all locations, r-axSpA patients showed more often bilateral entheseal lesions, which was only statistically significant for the os ischium: 6% vs 3% (*p* = 0.750) at the greater trochanter, 4% vs 1% (*p* = 0.253) at the lesser trochanter, 55% vs 34% (*p* < 0.001) at the os ischium, and 10% vs 4% (*p* = 0.145) at the iliac crest.

### Entheseal lesions and patient/disease characteristics

There was no significant correlation between the total number of radiographic entheseal lesions and the total number of tender entheses (rho = 0.051, *p* = 0.521). Interestingly, male r-axSpA patients showed significantly more radiographic entheseal lesions compared with female patients (median 2 vs 1, *p* < 0.005), whereas female r-axSpA patients showed significantly more tender entheses compared with male patients (median 5 vs 2, *p* < 0.001).

R-axSpA patients with radiographic entheseal lesions were significantly older (45.2 vs 35.1 years, *p* < 0.005) and had longer symptom duration than patients without lesions (18 vs 7.5 years, *p* < 0.005). Patients with BMI < 25 had lower total number of lesions than patients with BMI ≥ 25, with a trend towards statistical significance (16% vs 42%, *p* = 0.056). Especially enthesophytes were found significantly more often in r-axSpA patients with BMI ≥ 25 (65% vs 35%, *p* = 0.019).

R-axSpA patients with at least one radiographic entheseal lesion had significantly more spinal radiographic damage, illustrated by higher mSASSS total score (8.7 vs 1.0, *p* < 0.005) and cervical and lumbar mSASSS sub-scores (4.5 vs 0.5 and 4.0 vs 0.0, respectively, *p* < 0.005). Furthermore, r-axSpA patients with radiographic entheseal lesions had more often radiographic hip involvement defined by BASRI-hip score ≥ 2 than patients without entheseal involvement (9% vs 3%, *p* = 0.063). This difference was not statistically significant probably due to low number of hip involvement.

Univariable logistic regression analysis showed that male sex (OR 2.41; *p* = 0.021), age (OR 1.11; *p* < 0.001), symptom duration (OR 1.07; *p* = 0.003), and mSASSS total score (OR 1.08; *p* = 0.006) were significantly associated with the presence of any radiographic entheseal lesion. In multivariable analysis, symptom duration and mSASSS total score were independently associated with the presence of radiographic entheseal lesions (OR 1.06; *p* = 0.027 and OR 1.06; *p* = 0.049, respectively) (Table 3).

**Table 3:** Logistic regression analyses of patient characteristics and clinical assessments associated with the presence of radiographic entheseal lesions

	Univariable analysis			Multivariable analysis	
	OR (95% CI)	*P*-value	*R*^2^	OR (95% CI)	*P*-value
Male sex	2.41 (1.14–5.09)	0.021	0.048	1.71 (0.68–4.29)	0.250
Age	1.11 (1.07–1.17)	< 0.001	0.251		^†^
Symptom duration	1.07 (1.02–1.11)	0.003	0.100	1.06 (1.01–1.12)	0.027
HLA-B27 +	0.49 (0.18–1.38)	0.179	0.019		
BMI	1.05 (0.95–1.17)	0.337	0.012		
History of peripheral arthritis	0.77 (0.37–1.62)	0.487	0.005		
Tender entheses	1.02 (0.93–1.13)	0.679	0.002		
ASDAS	0.95 (0.61–1.50)	0.830	0.000		
BASDAI	1.02 (0.81–1.28)	0.863	0.000		
CRP	0.99 (0.97–1.01)	0.306	0.010		
mSASSS total score	1.08 (1.02–1.14)	0.006	0.173	1.06 (1.00–1.11)	0.049

*r-axSpA* radiographic axial spondyloarthritis, *OR* odds ratio, *CI* confidence interval, *n* number, *HLA-B27* + human leukocyte antigen B27 positive, *BMI* body mass index, *ASDAS* Axial Spondyloarthritis Disease Activity Score, *CRP* C-reactive protein, *BASDAI* Bath Ankylosing Spondylitis Disease Activity Index, *mSASSS* modified Stoke Ankylosing Spondylitis Spinal Score

^†^Age was not included in the multivariable model due to the strong association with duration of symptoms

## Discussion

Our study showed that radiographic entheseal lesions at the hip and pelvic region are observed frequently in both r-axSpA patients and age- and sex-matched controls, however, almost 3 times more often in r-axSpA patients (344 vs 124 lesions). All types of entheseal lesions were significantly more prevalent, and all entheseal locations were significantly more often affected in r-axSpA patients than in controls, with also more often bilateral involvement. Os ischii was the most frequently affected location, and erosions/cortical irregularities was the most prevalent type of lesion in both r-axSpA patients and controls.

Until now, little was known about radiographic entheseal lesions at the pelvic and hip region in axSpA. In a large multi-center study, pelvic radiographs were compared between 588 psoriatic arthritis patients and 536 patients with inflammatory arthritis, of which 13% had r-axSpA [26]. The lesions scored were entheseal erosions and irregular bony proliferation, which were found in 18% and 28% of patients in r-axSpA, compared with 6% and 13% in PsA, 7% and 10% in RA, and 3% and 3% in undifferentiated SpA, respectively. These differences were mainly due to entheseal erosions seen at the pelvic region and entheseal new bone formation at the os ischii. In our study, the number of erosions found in r-axSpA patients was higher compared with the study of Helliwell et al., which might be explained by scoring cortical irregularities together with erosions in our study. The prevalence of enthesophytes/new bone formation was comparable in both studies, 31% vs 28%, respectively. Although it is difficult to compare studies, because different lesions and patients groups have been evaluated, both studies show that radiographic entheseal lesions at the pelvic region are common in r-axSpA.

In our study, erosions/cortical irregularities were the most prevalent radiographic entheseal lesions observed in both r-axSpA patients and controls. In a study with 64 healthy subjects, erosions assessed with US were infrequently found, but none of the examined entheseal sites were located at the pelvis [13]. Although erosions can be observed with US, this assessment is better in evaluating inflammation, whereas radiographs are used to evaluate damage, which might explain the difference between our findings and the study of Guldberg-Møller et al.

In three studies evaluating inflammatory entheseal lesions with WB-MRI in axSpA, the pelvis was the most affected region [6, 11, 29]. Althoff et al. and Guo et al. looked at bone marrow edema and surrounding soft tissue edema and Krabbe et al. at entheseal osteitis and soft tissue inflammation. In the first study, 21% of the 42 axSpA patients had inflammatory entheseal lesions detected with WB-MRI at baseline, most frequently found at the pelvic region (55%) [11]. The iliac crest and lower sacrum were the most affected locations of the pelvic region. The second study found inflammatory entheseal lesions in 78% of 60 axSpA patients, and also, the pelvic region was most often affected (52%) [6]. The third study found inflammatory lesions at 10% of the evaluated entheseal locations in 49 axSpA patients, including the iliac crest, greater trochanter, and os ischium [29]. Entheseal involvement was frequently found at the greater trochanter (21%) and os ischium (16%). These WB-MRI studies are not comparable with our study, due to difference in imaging inflammatory versus structural lesions. Also, the patient populations were different; our patients were older (43 vs 33–36 years) and had longer symptom duration (16 vs 1–5 years). Disease activity in our patients was higher compared with the patients in the study of Guo et al. (mean ASDAS 2.7 vs 2.3). Furthermore, only one WB-MRI study used a control group. Guo et al. found inflammatory entheseal lesions in 32% of 50 control patients with chronic low back pain [6].

We found that specificity was highest for calcifications at any location and for erosions/irregularities at the greater trochanter. In general, specificity was rather low taken into account all the different types of entheseal lesions at all locations. This can be explained that most prevalent lesions (erosions/irregularities) were found at two of four pelvic locations; however, these were not very specific for r-axSpA. The other types of lesions have a high specificity for r-axSpA but are not very prevalent. Of the previously mentioned WB-MRI studies, Guo et al. found a specificity of 90% for inflammatory entheseal lesions at the pelvis.

In our study, male sex, older age, longer symptom duration, and more radiographic spinal damage were significantly associated with more radiographic entheseal lesions at the pelvic region in r-axSpA patients. Additionally, being overweight (BMI 25–30) or obese (BMI > 30) was associated with significantly more enthesophytes. These results are in line with results of US studies, which showed an association between the presence of entheseal lesions and older age, longer disease duration, more severe spinal damage, and higher BMI [15–17, 30]. Our finding that male r-axSpA patients showed more radiographic entheseal lesions compared with female r-axSpA patients is in line with previous studies regarding radiographic spinal damage [31]. Additionally, female patients experienced more tender entheses, which shows that the extent of experienced pain at entheseal sites is often not associated with the presence of entheseal structural damage. Therefore, clinical tender entheses are not equal to radiographic entheseal involvement or outcome in axSpA [31].

Systematic literature reviews in SpA showed that higher BMI is associated with more new bone formation and higher mSASSS [32, 33]. Also, in healthy individuals, it has been shown that both inflammatory and structural US entheseal lesions are associated with higher BMI, suggesting an additional effect of BMI on mechanical strain in overweight and obese axSpA patients [13, 14].

The relation between being overweight or obese and the presence of entheseal lesions highlight the importance to pay attention to lifestyle in axSpA. This is supported by a study in which weight loss in obese patients with PsA treated with a very low energy diet showed significant improvement of disease activity in clinically evaluated entheses with the Leeds enthesitis index [34]. Furthermore, obesity was found to be associated with poor clinical outcome, higher disease activity, and lower response to TNFi in patients with axSpA [32, 33, 35]. Therefore, attention should be paid to obesity and the guidance in weight loss in axSpA in daily clinical practice to improve clinical outcome such as disease activity including entheseal involvement and health-related quality of life.

Multivariable analysis showed that symptom duration and mSASSS total score were independently associated with the presence of radiographic entheseal lesions in r-axSpA, indicating that entheseal involvement is more present in patients with more longstanding and severe axial disease. This finding is in line with data from the French DESIR cohort, in which the presence of ≥ 1 enthesophyte on US structural damage score of 4 peripheral entheses was associated with higher mSASSS [16]. This indicates that clinicians should be aware for entheseal disease in patients with more severe spinal damage.

This was a first study regarding structural lesions at entheseal locations visible on pelvic radiographs in r-axSpA patients compared with age- and sex-matched controls. The evaluation of radiographic entheseal lesions in r-axSpA patients seems valid, feasible, and valuable. However, it should be kept in mind when interpreting the results of our study, only r-axSpA patients with active disease (before starting TNFi) were included. This selection of more severe r-axSpA patients may have resulted higher prevalence of radiographic entheseal lesions. Therefore, the results of our study cannot be extrapolated to the total axSpA population. Although controls were matched for age and sex based on frequency matching, unfortunately, we had no individual demographic data available for the controls, especially data on age and BMI would have been interesting. Due to privacy regulations, pelvic radiographs from controls from the emergency department could not be obtained with clinical files. Therefore, the indication for the pelvic radiograph was not known.

Due to the cross-sectional design of our study, we cannot provide information on progression of entheseal lesions over time nor on the predictive value of radiographic entheseal lesions on disease outcome. For further research, we therefore recommend longitudinal evaluation of pelvic radiological entheseal lesions in relation to the disease activity and spinal radiographic damage, the comparison of pelvic entheseal lesions between patients with radiographic and non-radiographic axSpA (irrespective of treatment), and exploring risk factors for pelvic radiographic entheseal involvement.

In conclusion, this cross-sectional study showed that radiographic entheseal lesions at the hip and pelvic region are significantly more prevalent in r-axSpA patients than in age- and sex-matched controls, with more often bilateral involvement. Erosions/cortical irregularities were most prevalent type of lesion, whereas enthesophyte and calcifications were more specific for r-axSpA. Additionally, longer symptom duration and more severe spinal radiographic damage are independently associated with the presence of radiographic entheseal involvement at the hip and pelvic region in r-axSpA. Therefore, in daily clinical practice, it is recommended to pay attention to radiographic entheseal lesions on pelvic radiographs of r-axSpA patients besides assessing structural damage of the sacro-iliac joints. The presence of radiographic entheseal lesions may also indicate more severe (axial) disease, which may contribute to treatment and management decisions.

## References

[1] López-Medina C, Molto A, Sieper J, Duruöz T, Kiltz U, Elzorkany B et al (2021) Prevalence and distribution of peripheral musculoskeletal manifestations in spondyloarthritis including psoriatic arthritis: results of the worldwide, cross-sectional ASAS-PerSpA study. RMD Open 7(1):e001450 33462157 10.1136/rmdopen-2020-001450 PMC7816910

[2] Mease PJ, Liu M, Rebello S, Hua W, McLean RR, Yi E et al (2020) Characterization of patients with axial spondyloarthritis by enthesitis presence: data from the Corrona Psoriatic Arthritis/Spondyloarthritis Registry. ACR Open Rheumatol 2(7):449–456 32627974 10.1002/acr2.11154 PMC7368134

[3] De Winter JJ, Paramarta JE, De Jong HM, Van De Sande MG, Baeten DL (2019) Peripheral disease contributes significantly to the level of disease activity in axial spondyloarthritis. RMD Open 5(1):e000802 30713720 10.1136/rmdopen-2018-000802 PMC6340525

[4] Mathew AJ, Glintborg B, Krogh NS, Hetland ML, Østergaard M (2022) Enthesitis in patients with psoriatic arthritis and axial spondyloarthritis—data from the Danish nationwide DANBIO registry. Semin Arthritis Rheum 1:52 10.1016/j.semarthrit.2021.12.012 35027245

[5] Palominos PE, de Campos APB, Ribeiro SLE, Xavier RM, Xavier JW, de Oliveira FB et al (2019) Correlation of enthesitis indices with disease activity and function in axial and peripheral spondyloarthritis: a cross-sectional study comparing MASES, SPARCC and LEI. Adv Rheumatol 59(1):23 31208465 10.1186/s42358-019-0066-8

[6] Guo Z, Li B, Zhang Y, Kong C, Liu Y, Qu J et al (2022) Peripheral enthesitis assessed by whole-body MRI in axial spondyloarthritis: distribution and diagnostic value. Front Immunol 23:13 10.3389/fimmu.2022.976800 PMC9446460 36081521

[7] Kehl AS, Corr M, Weisman MH. Review: enthesitis: new insights into pathogenesis, diagnostic modalities, and treatment. Vol. 68, Arthritis and Rheumatology. John Wiley and Sons Inc.; 2016. p. 312–22. 10.1002/art.39458 PMC5195265 26473401

[8] Maksymowych WP, Mallon C, Morrow S, Shojania K, Olszynski WP, Wong RL et al (2009) Development and validation of the Spondyloarthritis Research Consortium of Canada (SPARCC) Enthesitis Index. Ann Rheum Dis 68(6):948–953 18524792 10.1136/ard.2007.084244

[9] Heuft-Dorenbosch L, Spoorenberg A, van Tubergen A, Landewé R, van ver Tempel H, Mielants H, Dougados M, vander Heijde D (2003) Assessment of enthesitis in ankylosing spondylitis. Ann Rheum Dis 62(2):127–132. https://www.annrheumdis.com 10.1136/ard.62.2.127 PMC1754445 12525381

[10] Schett G, Lories RJ, D’Agostino MA, Elewaut D, Kirkham B, Soriano ER, et al. Enthesitis: from pathophysiology to treatment. Vol. 13, Nature Reviews Rheumatology. Nature Publishing Group; 2017. p. 731–41. 10.1038/nrrheum.2017.188 29158573

[11] Althoff CE, Sieper J, Song IH, Weiß A, Diekhoff T, Haibel H et al (2016) Comparison of clinical examination versus whole-body magnetic resonance imaging of enthesitis in patients with early axial spondyloarthritis during 3 years of continuous etanercept treatment. J Rheumatol 43(3):618–624 26834218 10.3899/jrheum.150659

[12] Zhang H, Liang J, Qiu J, Wang F, Sun L (2017) Ultrasonographic evaluation of enthesitis in patients with ankylosing spondylitis. J Biomed Res 31(2):162–169 28808198 10.7555/JBR.31.20160088 PMC5445219

[13] Guldberg-Møller J, Terslev L, Nielsen SM, Koenig MJ, Torp-Pedersen ST, Torp-Pedersen A et al (2019) Ultrasound pathology of the entheses in an age and gender stratified sample of healthy adult subjects: a prospective cross-sectional frequency study. Clin Exp Rheumatol 37(3):408–13 30620269

[14] Bakirci S, Solmaz D, Stephenson W, Eder L, Roth J, Aydin SZ (2020) Entheseal changes in response to age, body mass index, and physical activity: An ultrasound study in healthy people. J Rheumatol 47(7):968–972 32007938 10.3899/jrheum.190540

[15] Aydin SZ, Can M, Alibaz-Oner F, Keser G, Kurum E, Inal V et al (2016) A relationship between spinal new bone formation in ankylosing spondylitis and the sonographically determined Achilles tendon enthesophytes. Rheumatol Int 36(3):397–404 26442943 10.1007/s00296-015-3360-8

[16] Ruyssen-Witrand A, Jamard B, Cantagrel A, Nigon D, Loeuille D, Degboe Y et al (2017) Relationships between ultrasound enthesitis, disease activity and axial radiographic structural changes in patients with early spondyloarthritis: Data from DESIR cohort. RMD Open 3(2):e000482 28955496 10.1136/rmdopen-2017-000482 PMC5604709

[17] Polachek A, Cook R, Chandran V, Gladman DD, Eder L (2017) The association between sonographic enthesitis and radiographic damage in psoriatic arthritis. Arthritis Res Ther. 19(1):1–18 28810926 10.1186/s13075-017-1399-5 PMC5558768

[18] Seven S, Pedersen SJ, Østergaard M, Felbo SK, Sørensen IJ, Døhn UM et al (2020) Peripheral enthesitis detected by ultrasonography in patients with axial spondyloarthritis—anatomical distribution, morphology, and response to tumor necrosis factor-inhibitor therapy. Front Med 15:7 10.3389/fmed.2020.00341 PMC7381133 32766263

[19] Arends S, Brouwer E, van der Veer E, Groen H, Leijsma MK, Houtman PM et al (2011) Baseline predictors of response and discontinuation of tumor necrosis factor-alpha blocking therapy in ankylosing spondylitis: a prospective longitudinal observational cohort study. Arthritis Res Ther 13(3):1 10.1186/ar3369 PMC3218909 21689401

[20] Lukas C, Landewé R, Sieper J, Dougados M, Davis J, Braun J et al (2009) Development of an ASAS-endorsed disease activity score (ASDAS) in patients with ankylosing spondylitis. Ann Rheum Dis 68(1):18–24 18625618 10.1136/ard.2008.094870

[21] Garrett S, Jenkinson T, Whitelock H, Gaisford P, Calin A (1994Dec) A new approach to defining disease status in ankylosing spondylitis: the Bath Ankylosing Spondylitis Disease Activity Index. J Rheumatol 21(12):2286–2291 7699630

[22] Calin A, Garret S, Whitelock H, Kennedy LG, O’Hea J, Mallorie P et al (1994) A new approach to defining functional ability in ankylosing spondylitis: the development of the Bath Ankylosing Spondylitis Functional Index. J Rheumatol 21(12):2281–2285 7699629

[23] Creemers MC, Franssen MJ, MA, Hof, Gribnau FW, Van De Putte LB, Van Riel P (2005) Assessment of outcome in ankylosing spondylitis: An extended radiographic scoring system. Ann Rheum Dis 64(1):127–9 10.1136/ard.2004.020503 PMC1755183 15051621

[24] MacKay K, Brophy S, Mack C, Doran M, Calin A (2000) The development and validation of a radiographic grading system for the hip in ankylosing spondylitis: the Bath Ankylosing Spondylitis Radiology Hip Index. J Rheumatol 27(12):2866–72 11128678

[25] Voudouris KP, Sidiropoulos P, Vounotrypidis P, Arvanitakis M (2003) Introduction Enthesial fibrocartilage-bone interaction: a radiographic study of selected sites of nonsynovial peripheral enthesopathy. J Musculoskel Neuron Interact 3:89–100 15758371

[26] Helliwell PS, Porter G, Lassere M, Rappo J, Mielants H, Van De Berghe M et al (2007) Sensitivity and specificity of plain radiographic features of peripheral enthesopathy at major sites in psoriatic arthritis. Skeletal Radiol 36(11):1061–1066 17849113 10.1007/s00256-007-0376-5

[27] Eshed I, Bollow M, McGonagle DG, Tan AL, Althoff CE, Asbach P et al (2007) MRI of enthesitis of the appendicular skeleton in spondyloarthritis. Ann Rheum Dis 66:1553–9 17526551 10.1136/ard.2007.070243 PMC2095313

[28] Herregods N, Dehoorne J, Pattyn E, Jaremko JL, Baraliakos X, Elewaut D et al (2015) Diagnositic value of pelvic enthesitis on MRI of the sacroiliac joints in enthesitis related arthritis. Pediatr Rheumatol 13(1):1–9 10.1186/s12969-015-0045-5 PMC4641332 26554668

[29] Krabbe S, Eshed I, Sorensen IJ, Jensen B, Moller JM, Balding L et al (2020) Whole-body magnetic resonance imaging inflammation in peripheral joints and entheses in axial spondyloarthritis: distribution and changes during adalimumab treatment. J Rheumatol 47(1):50–58 30936290 10.3899/jrheum.181159

[30] Wink F, Bruyn GA, Maas F, Griep EN, Van Der Veer E, Bootsma H et al (2017) Ultrasound evaluation of the entheses in daily clinical practice during tumor necrosis factor-α blocking therapy in patients with ankylosing spondylitis. J Rheumatol 44(5):587–593 28298566 10.3899/jrheum.160584

[31] Rusman T, van Vollenhoven RF, van der Horst-Bruinsma IE. Gender differences in axial spondyloarthritis: women are not so lucky. Vol. 20, Current Rheumatology Reports. Current Medicine Group LLC 1; 2018. 10.1007/s11926-018-0744-2 PMC5949138 29754330

[32] Zurita Prada PA, Urrego Laurín CL, Guillén Astete CA, Kanaffo Caltelblanco S, Navarro-Compán V. Influence of smoking and obesity on treatment response in patients with axial spondyloarthritis: a systematic literature review. Vol. 40, Clinical Rheumatology. Springer Science and Business Media Deutschland GmbH; 2021. p. 1673–86. 10.1007/s10067-020-05319-6 32880827

[33] Liew JW, Huang IJ, Louden DN, Singh N, Gensler LS (2020) Association of body mass index on disease activity in axial spondyloarthritis: systematic review and meta-analysis. RMD Open 6(1):e001225 32434828 10.1136/rmdopen-2020-001225 PMC7299511

[34] Klingberg E, Bilberg A, Björkman S, Hedberg M, Jacobsson L, Forsblad-D’Elia H, et al. 2019 Weight loss improves disease activity in patients with psoriatic arthritis and obesity: an interventional study. Arthritis Res Ther 21(1) 10.1186/s13075-019-1810-5 PMC6330463 30635024

[35] Maas F, Arends S, Van Der Veer E, Wink F, Efde M, Bootsma H et al (2016) Obesity is common in axial spondyloarthritis and is associated with poor clinical outcome. J Rheumatol 43(2):383–387 26669924 10.3899/jrheum.150648

